# Development of Fortified Citrus Olive Oils: From Their Production to Their Nutraceutical Properties on the Cardiovascular System

**DOI:** 10.3390/nu12061557

**Published:** 2020-05-27

**Authors:** Lorenzo Flori, Monica Macaluso, Isabella Taglieri, Chiara Sanmartin, Cristina Sgherri, Marinella De Leo, Valerio Ciccone, Sandra Donnini, Francesca Venturi, Luisa Pistelli, Alma Martelli, Vincenzo Calderone, Lara Testai, Angela Zinnai

**Affiliations:** 1 Department of Pharmacy, University of Pisa, Via Bonanno Pisano 6, 56126 Pisa, Italy; lorenzo.flori@phd.unipi.it (L.F.); marinella.deleo@unipi.it (M.D.L.); luisa.pistelli@unipi.it (L.P.); alma.martelli@unipi.it (A.M.); vincenzo.calderone@unipi.it (V.C.); angela.zinnai@unipi.it (A.Z.); 2 Department of Agriculture, Food and Environment (DAFE), University of Pisa, Via del Borghetto 80, 56124 Pisa, Italy; monica.macaluso@phd.unipi.it (M.M.); isabella.taglieri@for.unipi.it (I.T.); cristina.sgherri@unipi.it (C.S.); francesca.venturi@unipi.it (F.V.); 3 Interdepartmental Research Center “Nutraceuticals and Food for Health”, University of Pisa, Via del Borghetto 80, 56124 Pisa, Italy; 4 Department of Life Science, University of Siena, Via Aldo Moro 2, 53100 Siena, Italy; ciccone3@student.unisi.it (V.C.); sandra.donnini@unisi.it (S.D.); 5 Toscana Life Sciences Str. del Petriccio e Belriguardo 35, 53100 Siena, Italy

**Keywords:** extra virgin olive oil, cryogen, *Citrus* genus, phytochemical, nutraceutical value, *Citrus x aurantium*, *Citrus limon*, cardiovascular protection

## Abstract

Recently the use of food by-products as natural sources of biologically active substances has been extensively investigated especially for the development of functional foods fortified with natural antioxidants. Due to their content of bioactive compounds, such as carotenoids, flavonoids and limonoids, citru*s* peels could be suitable to formulate enriched olive oils able to boost healthy nutrition. The aim of this study was: (i) to determine the compositional and sensory profiles of citrus olive oil; and (ii) to evaluate its nutraceutical properties in rats with high fat diet-induced metabolic syndrome and oxidative stress. The results obtained show the potential of using citrus peels as a source of bioactive compounds to improve the sensory profile as well as the phytochemical composition of olive oil. We demonstrated that the production system of *Citrus x aurantium* olive oil and *Citrus limon* olive oil improves its organoleptic properties without altering its beneficial effects, which, like control extra virgin olive oil, showed protective effects relating to glucose and serum lipid levels, metabolic activity of adipocytes, myocardial tissue functionality, oxidative stress markers and endothelial function at blood vessel level.

## 1. Introduction

The *Citrus* genus (belonging to Rutaceae family) provides popular fruits worldwide consumed as fresh products and used for production of fruit juices. Further, its by-products are a source of important bioactive compounds with potential for animal feed, manufactured foods and health care [1]. 

*Citrus* fruits, including sour orange (*Citrus x aurantium* L.) and lemon (*C. limon*), (Osbeck), represent an important source of pharmacologically active secondary metabolites, mainly produced by the plants as self-defense against insects and microbial attacks [2]. 

Due to their phytochemical content, *Citrus* fruits exhibit interesting properties in health protection and disease prevention. They contain high levels of flavonoids, carotenoids and limonoids. Of note, approximately 95% of flavonoids are represented by flavanones, in particular naringenin, hesperetin and eriodictyol [3]. Epidemiological, clinical and preclinical evidence point out their nutraceutical benefits on the cardiovascular system, indeed vaso-relaxing and cardioprotective effects, observed with fruit juices and with purified *Citrus* flavanones [3,4,5,6,7]. Recently, naringenin and hesperetin have been demonstrated to be endowed with SIRT1-mediated anti-ageing properties, able to increase the life-span in nematode and yeast models [8,9,10,11,12]. Similar results have been reported with bergamot juice or other *Citrus* fruits [9]. Furthermore, a number of studies suggest that *Citrus* fruits contribute to cardiovascular health preservation through an improvement of cardiometabolic profile, reducing total and low-density lipoprotein (LDL) cholesterol and triglycerides in plasma and restricting the body weight gain associated with a high fat diet [13,14,15]. Moreover, epidemiological and clinical studies suggest that consumption of flavonoid rich foods could potentially improve human health and well-being, due to their protective effect against degenerative diseases [16]. 

*Citrus* fruits are also a good carotenoid source; indeed at least 110 different carotenes and xanthophylls have been detected [17]. These pigments, responsible for the color of peel and pulp of many *Citrus* fruits, are very important components of photosynthesis and are involved in preventing photo oxidation [2,17]. Carotenoids are well known for their vitamin action (vitamin A) as well as for providing positive health effects and reducing the risk of diseases, namely tumors, heart and ophthalmological disease [2,18].

Limonoids are another significant class of biologically active compounds in *Citrus*, especially in *C. mitis* and *C. sinensis*, that through stimulation of the enzyme glutathione S-transferase (GST) have anticancer effects [2] as well as other pharmacological properties such as antimicrobial, antioxidant, antidiabetic and insecticidal effects [19]. 

Within this framework, medical and public communities are considerably focusing their attention on foods containing bioactive compounds, therefore there is an increasing interest in formulating healthy functional foods enriched with nutraceutical compounds useful for the prevention of chronic diseases [20,21]. Fortified food (FF) is indeed one of the most effective tools to improve nutritional intake for several reasons: (i) FF could be available to many people by selecting the most appealing food form; (ii) FF is sociably acceptable since people do not have to opt for change in food characteristics or diet; (iii) FF could be rapidly introduced and its advantages are rapidly attained; (iv) FF are generally thought riskless and cost-effective [22].

On this basis, recently Zinnai’s group produced, by means of an innovative method, fortified olive oils in which peels or leaves of *Citrus* (*C. aurantium* or *C. limon*) were cryomacerated and pressed with olive fruits [23,24] in order to obtain a food enriched with nutraceutical value typical of *Citrus* fruits. The addition to olives of *Citrus* cryomacerated by-products (peels or leaves) during the olive oil extraction process enables the production of oils with a high level of biologically active substances. In particular, the specific composition as well as the sensorial properties of the obtained oil varied as a function of the considered fortifying agent. Indeed, the organoleptic profile of the fortified oils was profitably improved in terms of smell complexity and hedonic response, when compared with the control [23,24]. 

The aim of this research was: (i) to determine the compositional and sensory profiles of *Citrus* olive oils (COOs); and (ii) to evaluate their nutraceutical properties in rats with high fat diet-induced metabolic syndrome and oxidative stress.

## 2. Materials and Methods 

### 2.1. Plant Material

*Citrus x aurantium* and *C. limon* fruits were collected at full maturity during crop season 2018/2019 in the *horti simplicium* of the monks of the Charterhouse of Pisa (Certosa di Pisa, located in Calci), where they are not subjected to any agronomic treatment. The olives (Moraiolo cv; Leccino cv) were collected during crop season 2018/2019 and supplied by an organic private Tuscan farm (Azienda Agricola Val Di Lama, Pontedera (PI), Italy) and characterized following exactly the procedure reported by Venturi and colleagues. [25] (Table 1). 

### 2.2. Citrus Peels Cryomaceration and Citrus Olive Oil Extraction

*Citrus* fruits were manually peeled (total average thickness of periderm layer: 2 mm) using a ceramic blade to prevent the initiation of oxidative processes, according to a previous paper [25].

To avoid the oxidation of the bioactive compounds present in the peels and maximize their extraction, the peels stayed in contact with dry ice (CO_2,s_) in equiponderal quantities (ratio 1:1) for 24 h [25].

After cryomaceration, peels were directly added to olives (25% w/w) before milling in order to obtain the COOs (Table 2). 

The olive oil extraction was performed by means of a micro olive oil mill (Spremioliva mod. C30, Mori-TEM srl, Italy). The working conditions and the technical features of the micro oil mill were described in a previous paper [23].

### 2.3. Citrus Oils Chemical Analyses 

#### 2.3.1. Chemicals

Acetic acid, ethanol, sodium carbonate, ethoxyethane, iso-octane, chlorane 37.0%, sodium hydroxide 0.1 N, sodium thiosulphate 0.01 N, potassium iodide, starch indicator solution 1.0%, ABTS (2,2′-azinobis(3-ethylbenzothiazoline-6-sulphonic acid)), 4-(2-Hydroxyethyl)phenol, Trolox (6-Hydroxy-2,5,7,8-tetramethylchroman-2-carboxylic acid), TrisHCl (2-amino-2-(hydroxymethyl)propane-1,3-diol chlorane) and Lithium perchlorate (LiClO_4_) were supplied by Sigma Aldrich (Milan, Italy). 3,4,5-trihydroxybenzoic acid was purchased from Carlo Erba (Milan, Italy). 3,3-bis(4-hydroxyphenyl)-2-benzofuran-1(3*H*)-one 1% and Folin–Ciocalteau reagent were obtained from Titolchimica (Pontecchio Polesine, Italy).

Methanol, *n*-hexane, and formic acid for HPLC analyses were purchased from VWR (Milan, Italy). HPLC grade water (18 mΩ) was obtained by a Mill-Ω purification system (Millipore Corp., Bedford, MA, USA). 

CelLytic™ MT Cell Lysis Reagent was obtained from Life Technologies (Carlsbad, CA, USA). Streptavidin-conjugated HRP,3,3-diaminobenzidine tetrahydrocloride (DAB), Eukitt^®^ quick-hardening mounting medium fibrinogen, thrombin and VEGF were from Merck KGaA (Darmstadt, Germany). Tissue-Tek O.C.T. was from Sakura (San Marcos, CA, USA). Anti-COX-2 was from Origene (Rockville, MD, USA) [TA313292]; anti-ALDH1A1 and anti-4-HNE were from Abcam (Cambridge, UK) [ab9883 and ab46544]; anti-Catalase and anti-β-actin were from Merck KGaA (Darmstadt, Germany) [C0979 and A2228]; anti-mPGES-1 was from Cayman Chemical (Ann Arbor, MI, USA) [160140]; anti-iNOS, anti-eNOS, p65, p22-phox and anti-CD40 were from Santa Cruz Biotechnology (Dallas, TX, USA) [sc-7271, sc-8008, sc-271968 and sc-975].

#### 2.3.2. Chemical Quality Standards

The quality parameters defined in trade standards (free acidity, peroxide index and spectrophotometric indices) were measured according to the official analytical methods thoroughly described in European legal prescription [26]. 

#### 2.3.3. Total Phenol 

Total phenol (TP) was extracted by a liquid–liquid extraction with a solution of methanol:water (80:20 v/v) according to a previous work [20], then the extracts were stored under inert atmosphere at −20 °C until use. TP concentration was measured by Folin-Ciocalteau colorimetric assay slightly modified: briefly, TP extract (1 mL), Folin−Ciocalteu reagent (1 mL), and 7.5% sodium carbonate (9 mL) and deionized water (14 mL), were added to a 25 mL glass flask, mixed, and, after 120 min of incubation at room temperature, absorbance of the samples was measured at 765 nm against a blank. TP content was expressed using a calibration curve prepared with gallic acid as the standard [27].

#### 2.3.4. Free-Radical Scavenging Capacity (FRSC)

FRSC was determined using both the DPPH (2,2-diphenyl-1-picrylhydrazyl) assay (FRSC_DPPH_) [28] and the ABTS (2,2’-azino-bis(3-ethylbenzothiazoline-6-sulfonic acid)) assay (FRSC_ABTS_) [29]. The radical solution was prepared according to the procedure described by Fellegrini [30], while the Trolox dose–response curve used was in the 0.2–1.5 mM range. FRSC was calculated as Trolox Equivalent Antioxidant Capacity (TEAC) per mL of extract [24].

#### 2.3.5. Intensity of Bitterness (IB)

The intensity of bitterness was determined following the method developed by Gutiérrez Rosales et al. [30] without modifications. Extraction of bitter compounds was performed in methanol:water (1:1) from 1.00 ± 0.01 g of samples using 6 mL extraction columns (Sep-Pak C18 Classic Cartridge, Waters s.p.a., Sesto San Giovanni (MI), Italy) and absorbance of extract was measured at 225 nm (K225).

#### 2.3.6. Carotenoids and Chlorophylls

Total carotenoids and chlorophylls were evaluated from the absorption spectrum of the pigment extract in cyclohexane, at 470 and 670 nm respectively, according to the procedure reported by Minguez Mosquera without modifications [31].

#### 2.3.7. Extraction and Detection of Tocopherols (Vitamin E)

Tocopherols were extracted in the dark as reported in the literature [32,33]. In particular, α-, β-, γ-, and δ-tocopherol isoforms were determined by isocratic RP-HPLC (Shimadzu apparatus model LC-20AD; electrochemical detector Metrohm model 791, Varese, Italy; glassy carbon electrode) and LC Solution software (Shimadzu) for the integration of peaks [34]. Detection was performed at +0.6 V and at 25 °C, with a Nova Pak C°18 4 μm column (3.9 × 150 mm) [34,35]. The extracts were eluted with 95% methanol containing 20 mM LiClO_4_ at a flow rate of 1 mL/min. For the identification and quantification of peaks, a calibration curve was prepared using standard mixtures of α-, β-, γ-, and δ-tocopherol provided by Sigma (Milan, Italy) in the range of 25 to 75 ng [34].

#### 2.3.8. HPLC-PDA/UV-ESI-MS/MS Analyses of Olive Oil Samples

All olive oil samples were subjected to the same extraction procedure in order to obtain their polar fraction. Two mL of each sample were first dissolved in 2 mL of *n*-hexane, then extracted with 4 mL of MeOH-H_2_O 70% v/v shaking with a vortex mixer for 3 min. The suspensions were kept for 30 min at room temperature, then centrifuged for 10 min at 1145× *g*. Each hydroalcoholic phase was extracted again with 2 mL of *n*-hexane using a vortex mixer for 3 min, then left for 30 min at room temperature, and centrifuged (10 min at 1145× *g*). The hydroalcoholic extracts of each sample were finally dried, weighed and dissolved in MeOH at a final concentration of 2.0 mg/mL for injection in the LC-MS system.

The HPLC-PDA/UV-MS equipment was composed of a Surveyor autosampler and a Surveyor LC pump, interfaced with a Surveyor PDA/UV detector and a LCQ Advantage ion trap mass spectrometer (ThermoFinnigan, San Jose, CA, USA). The phenolic extracts (20 μL) were eluted on a Synergi Fusion-RP column, 4.6 × 150 mm, 4 μm particle size (Phenomenex, Milan, Italy) using a mixture of methanol (solvent A) and formic acid in water 0.1% v/v (solvent B), at flow rate of 0.8 mL/min with a splitting system of 2:8 to MS detector (160 μL/min) and PDA detector (640 μL/min), respectively. A linear gradient increasing 5% to 70% A was developed within 65 min. PDA/UV data were registered in a wavelength range of 200–600 nm, with 254, 280, and 325 nm as preferential channels. The ESI interface was used in negative ion mode and molecules were detected in a scan range of *m*/*z* 150–2000. Ionization conditions were optimized as previously reported [36]. 

#### 2.3.9. Statistical Analysis of Chemical Data

Data are expressed as the means of three independent experiments. The significance of differences among means was determined by one-way ANOVA (CoStat, Version 6.451, CoHort Software, Pacific Grove, CA, USA). Comparisons among means were performed by the Tukey’s test (*p* < 0.05). 

Xcalibur 3.1 software was used for the analyses of UV and MS data.

### 2.4. Sensory Analysis

#### 2.4.1. Quantitative Descriptive Analysis

Ten trained assessors (six women and four men, age range 23–60 years), members of the expert panel of the Department of Agriculture, Food and Environment (DAFE) of the University of Pisa, performed the sensory assessment of samples.

**Figure 1:**
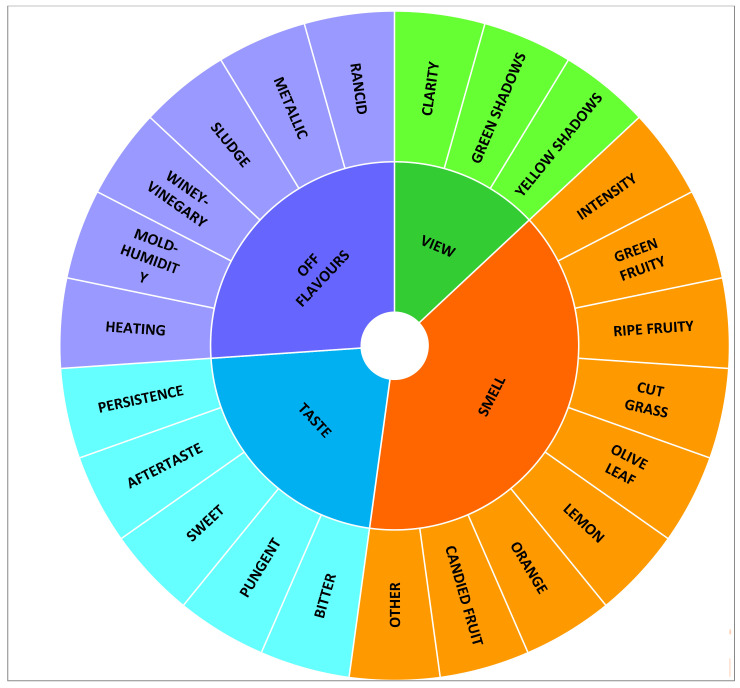
Sensory wheel developed for the descriptive evaluation of *Citrus* olive oils.

Before the tasting sessions, a preliminary and specific training session was arranged for the assessors [37], aimed not only at panel training but also to design the optimal method for the sensory evaluation of *Citrus* olive oils. Thereafter, the trained panel was involved in a consensus panel focused on the selection and definition of the proper descriptors. Starting from the set of quantitative parameters previously developed during previous works [23,24], a final set of 23 attributes for *Citrus* olive oil assessment was individuated by agreement among panelists (Figure 1).

Tasting trials were performed under the conditions described by [23,24]; each panelist rated the intensity of each parameter on a scale from 0 (no perception, minimum) to 9 (maximum), using a technical evaluation sheet based on the sensory wheel depicted in Figure 1.

#### 2.4.2. Statistical Analysis of Sensory Data

The sensory wheel was created using XLSTAT version 2019.4.1 (Addinsoft Inc., 244 Fifth Avenue, Suite E100, New York, NY 10001, USA). Sensory data were treated using the statistical software Big Sensory Soft 2.0 (ver. 2018).

### 2.5. In Vivo Evaluation of Nutraceutical Value of Olive Oils

In vivo experiments were carried out according to European (EEC Directive 2010/63) and Italian (D.L. 4 March 2014 n.26) legislation (number protocol 144/2019-PR, 18/02/2019); moreover, ARRIVE guidelines have been put into practice. ARRIVE guidelines are specific recommendations for in vivo research, aimed at improving the quality of scientific dissemination and assuring a reproducibility of animal tests [38]. Animals were housed in cages with food and water *ad libitum*, and they were exposed to a 12 h dark/light cycle. The experiments were conducted on adult male Wistar rats (3-4 months old, ENVIGO) with a body weight between 342 g and 350 g. In order to avoid hormonal interferences, we have chosen to carry out the experiments only on male rats. The number of animals per group has been established by using the statistical program G*Power.

The animals were randomly divided into five groups (five animals per group) and treated for 21 days: first group was treated with a standard diet (STD, ENVIGO; for composition see Table 3; polyphenol free pellets were used); second group was treated with a high fat diet (HFD, SAFE; for composition see Table 3). 

Third group was treated with HFD + extra-virgin olive oil (EVOO) 5.5% p/p (HFD + EVOO) mixed in powder feed. The amount of EVOO was calculated on the basis of daily intake of polyphenols suggested by the regulatory authorities [39]. Fourth group was treated with HFD + *Ca*OO 5.5% p/p (HFD + *Ca*OO) mixed in powder feed. Fifth group was treated with HFD + *Cl*OO 5.5% p/p (HFD + *Cl*OO) mixed in powder feed. Water intake was evaluated daily, while body weight and the food intake were measured three times a week for each animal; however, supplementations were administered daily on the basis of different experimental groups. After three weeks, at the end of the treatment, each animal was deprived of food and after 24 h was anesthetized with Thiopental Sodium (70 mg/kg, MSD animal health). Blood pressure was measured using a sphygmomanometric (non-invasive) method commonly called Tail Cuff. The blood was collected, through the caudal vein, to perform a blood glucose test (Glucocard G meter, Menarini Diagnostics). Subsequently the animals were sacrificed with an overdose of Thiopental Sodium. Blood was collected in tubes with the anticoagulant EDTA (BD Vacutaine) by intracardiac puncture, and organs (heart, aorta, liver, abdominal adipose tissue, and brown adipose tissue) were removed, weighed, and stored for functional, enzymatic and biochemical investigations. 

Blood was immediately used for the evaluation of lipid panel (total, high-density lipoprotein (HDL) and LDL cholesterol and triglycerides) by using Cobas b101 instrument (Roche Diagnostics). Blood samples were centrifuged at 3200 RPM for 10 min, obtaining plasma, which was frozen at −20 °C for subsequent analysis. 

Blood samples were then collected from 5 animals per group and results were expressed as mean ± SEM. Cardiovascular risk was calculated as ratio between total cholesterol and HDL cholesterol levels. One-way ANOVA followed by Bonferroni’s post hoc test was used to compare groups for statistical differences (*p* < 0.05).

#### 2.5.1. Functional Analysis of Cardiac Mitochondrial Membrane Potential

Hearts deriving from animals 21 days-treated were cut into small 2/3 mm^3^ pieces in ice cold isolation buffer (composition: Sucrose 250 mM, Tris 5 mM, EGTA 1 mM; pH 7.4). The pieces were homogenized with Ultra-Turrax (model: IKA, T-18 Basic). After three homogenization cycles of about 20 s, the first centrifugation was carried out: 1090× *g*, 3 min, 4 °C. The supernatant obtained was centrifuged at 11,970× *g*, 10 min, 4 °C. The pellet was preserved and resuspended in an isolation buffer without EGTA and centrifuged using the previous conditions. The pellet obtained, corresponding to the mitochondrial fraction, was resuspended in 400 μL and transferred to a frozen eppendorf. The mitochondrial protein concentration in the supernatant was determined spectrophotometrically by Bradford assay (Bio-Rad, Hercules, CA, USA), using a microplate reader (EnSpire, PerkinElmer, Waltham, MA, USA).

The membrane potential (∆Ψm) of the isolated mitochondria was determined using a potentiometric method. The lipophilic cation tetraphenylphosphonium (TPP^+^), used for this procedure, was detected with a TPP^+^ sensitive mini electrode (WPI, TipTPP, Sarasota, FL, USA), coupled to a reference electrode (WPI, Sarasota, FL, USA) and using data acquisition software (BiopacInc, Goleta, CA, USA). Mitochondria (1 mg protein/mL) were then added to the incubation buffer (composition: KCl 120 mM, K_2_HPO_4_ 5 mM, Hepes 10 mM, Succinic Acid 10 mM, MgCl_2_ 2 mM, TPP^+^Cl^−^ 10 μM; pH 7.4), and continuously stirred with a small magnet to generate a mitochondrial suspension.

The membrane potential value was calculated according to the following experimental equation derived from Nernst Equation (1):

(1) $$\Delta \Psi_{m}=60\;\times \;\log\frac{\frac{V_{0}{\left[{\mathrm{TPP}}^{+}\right]}_{0}}{{\left[{\mathrm{TPP}}^{+}\right]}_{t}}-V_{t}-K_{0}P}{V_{m}P+K_{i}P}$$

where ∆Ψm is the mitochondrial membrane potential (mV), V_0_ is the volume of the incubation medium before the addition of mitochondria, V_t_ is the volume of the incubation medium after the addition of the mitochondria, V_m_ is the volume of the mitochondrial matrix (taken as 1 µL/mg protein), [TPP^+^]_0_ and [TPP^+^]_t_ represent, respectively, the TPP^+^ concentrations recorded before addition and at time t, P is the protein concentration expressed in mg, and K_0_ and K_i_ are apparent external and internal partition coefficients of TPP^+^ (14.3 μL/mg and 7.9 μL/mg, respectively). The mitochondrial membrane potential was evaluated on five different animals per group, and data were expressed as mean ± SEM. One-way ANOVA followed by Bonferroni’s post hoc test was used to compare groups for statistical differences (*p* < 0.05).

#### 2.5.2. Evaluation of Citrate Synthase Activity on Adipose Tissue 

Frozen adipose tissues deriving from 21 days-treated rats were homogenized on ice with an ultra-turrax homogenizer (IKA-Werke GmbH & Co., Staufen, Germany) in a cold buffer (composition: Sucrose 250 mM, Tris 5 mM, EGTA 1 mM, Triton X-100 0.02%; pH 7.4). Homogenates were then at 12,000× *g* for 15 min at 4 °C (EuroClone, Speed Master 14 R centrifuge, Milan, Italy). The supernatant was used for determination of the citrate synthase activity, and the protein concentration in the supernatant was determined spectrophotometrically by Bradford assay (Bio-Rad, Hercules, CA, USA), using a microplate reader (EnSpire, PerkinElmer, Waltham, MA, USA). Proteins were then diluted in Tris-buffer 100 mM (pH 8.2) containing 5,5′-dithiobis-(2-nitrobenzoic) acid (DTNB, 100 µM) and acetyl-coenzyme A (100 µM). The assay was performed in 96 multi-well plates (1 µg of proteins per well) and the reaction was initiated by addition of oxaloacetic acid 500 µM. The absorption of the reaction product was measured spectrophotometrically at 30 °C and 412 nm every 30 s for 15 min. Citrate synthase activity was determined by comparing the activity in the samples to that of known concentrations of the isolated enzyme (Sigma-Aldrich, St. Louis, MO, USA). 

The assay was performed on five animals per group. Citrate synthase activity was expressed in mU/µg protein. Data were analyzed by a computer fitting procedure (software: GraphPad Prism 5.0). Results were expressed as mean ± SEM. One-way ANOVA followed by Bonferroni’s post hoc test was used to compare groups for statistical differences (*p* < 0.05).

#### 2.5.3. Western Blot

Western blot was performed on tissue samples as described previously [40]. Samples were maintained in CelLytic™MT supplemented with 2 mM Na_3_VO_4_ and 1x Protease inhibitor cocktail for mammalian cells (Sigma Aldrich, St. Louis, MO USA). Protein extraction from aortas started with disruption and homogenization using the TissueLyser II (#85300 Qiagen, Germantown, MD, USA). Samples were frozen/unfrozen twice in liquid nitrogen and then sonicated on ice for a total of 2 min, with a 15 s run and 15 s pause to limit sample heating. Cell lysates were centrifuged at 16,000× *g* for 20 min at 4 °C and the supernatants were then collected. Protein concentration was determined using the BCA protein assay kit (#23227 Thermo Fisher Scientific, Rockford, IL, USA). Electrophoresis (50 μg of protein/sample) was carried out in 4–12%Bis-Tris Gels (Life Technologies, Carlsbad, CA, USA). Proteins were then blotted onto nitrocellulose membranes, incubated overnight with primary antibodies, and then detected by enhanced chemiluminescence system (Biorad, Hercules, CA, USA). Results were normalized to those obtained by using an antibody against β-actin. All experiments were performed at least three times. Immunoblots were analyzed by densitometry using NIH Image J 1.48v software, and the results, expressed as arbitrary density units (ADU) ± SD, were normalized to β-actin. One-way ANOVA followed by Bonferroni’s post hoc test was used to compare groups for statistical differences (*p* < 0.05).

#### 2.5.4. Tube Formation from Vessel Rings

Vessel sprouting form rat aorta was evaluated as described previously [41].

Rat aortas were isolated in sterile conditions, and perivascular fibrous tissue was removed under a dissecting microscope. Rings 1–2 mm long were produced and rinsed in serum-free medium (DMEM4500; Euroclone, Milan, Italy). Vessel fragments were then included in a fibrin gel obtained by adding 400 μL of a human fibrinogen solution (3 mg/mL in DMEM4500 medium) into each well of 24-multiwell plates. Once vessel rings were positioned in the well with the lumen oriented horizontally in the center of the solution, gelation of the fibrinogen was induced with bovine thrombin (15 units/mL). After 20 min, 400 μL of EGM-2 medium was added with antibiotics (100 units/mL penicillin and 100 μg/mL streptomycin) and 10% FBS were added to each well. After 24 h, the medium was removed, the gels washed, and substances were added to the medium supplemented with VEGF (25 ng/mL). The organ culture was kept at 37 °C, 5% CO_2_. Each experimental point was run in triplicate.

Quantitative evaluation of newly formed structures was carried out at day 3. Wells were observed with an inverted microscope and photographed. The sprouting was evaluated from three vessel rings from at least three different animals per group, and data were expressed as vessel length ± SD. One-way ANOVA was used to compare groups for statistical differences (*p* < 0.05).

#### 2.5.5. RNA Isolation and Quantitative RT-PCR

Samples were maintained in CelLytic™MT supplemented with 2 mM Na_3_VO_4_ and 1x Protease inhibitor cocktail for mammalian cells (all reagents were from Sigma Aldrich). RNA isolation and quantitative RT-PCR (qRT-PCR) were performed on tissue samples. RNA extraction from tumor samples started with disruption and homogenization using the TissueLyser II (#85300 Qiagen). Total RNA was prepared using RNeasy Plus Kit (#74134 Qiagen) following manufacturer’s instructions. One μg RNA was reverse transcribed using QuantiTect Reverse Transcription Kit (#205313 Qiagen) and quantitative RT-PCR was performed using QuantiNovaSYBR Green PCR Kit (#208056 Qiagen) in a Rotor-Gene qPCR machine (Qiagen). Fold change expression was determined by the comparative *C*_t_ method (ΔΔ*C*_t_) normalized to GAPDH expression. qRT-PCR data are represented as fold change relative to STD group, which was assigned to 1, and expressed as mean ± SD. One-way ANOVA was used to compare groups for statistical differences (*p* < 0.05).

#### 2.5.6. Immunohistochemical Analysis

Seven-μm-thick cryostat sections from tissue samples were used for immunohistochemical staining with anti-CD40 antibody. Cryostat sections were first fixed in 4% paraformaldehyde (PFA) for 20 min and incubated for 10 min in 3% H_2_O_2,_ washed (3 × 5 min) in PBS (without Ca^++^ and Mg^++^) and then incubated with goat serum 5% for blocking background staining. Rabbit monoclonal anti-CD40 antibody diluted 1:100 in PBS with 0.05% goat serum was applied for 18 h at 4 °C. Sections were than washed (3 × 5 min in PBS, 0.05% goat serum) and incubated for 60 min in the appropriate species–specific biotinylated secondary antibodies (goat anti rabbit IgG). Following washing (3 × 5 min in PBS, 0.05% goat serum), the sections were incubated for 10 min in streptavidin-conjugated HRP. After this incubation, sections were exposed to 3,3-diaminobenzidine tetrahydrocloride (DAB, detection kit, Millipore, Milan, Italy) for 8 min to produce a brown reaction product. Sections were then counterstained in hematoxylin and mounted in Eukitt^®^ Quick-hardening mounting medium.

## 3. Results

### 3.1. Oil Chemical Characterization 

The characterization of *Ca*OO and *Cl*OO obtained by means of the addition of *Citrus* peels to olives during the oil extraction has been performed against the control (CEVOO), taking into account not only the quality criteria specified in the trade standard, but also the phytochemical features due to the detected bioactive compounds.

#### 3.1.1. Legal Quality Parameters

The quality parameters defined in the trade standard aim to classify oil into each of the different definitions [42]. As shown in Table 4, even though the milled olives were quite ripe, with a maturity index of 4.1 on a scale of 7.0 (see Section 2.1, Table 1), the olive oil samples were characterized by legal quality parameters (free acidity, peroxide index and spectrophotometric values indicated as K_232_, K_270_ and ∆K) lower than the maximum limits established by EU Regulation [26] for extra-virgin olive oil (EVOO).

Free acidity range was from 0.60 (*Ca*OO) to 0.74 % of oleic acid (*Cl*OO and CEVOO), probably because of the high ripeness degree of the raw material. The peroxide index was always below 10 mEq O_2_/kg oil, indicating a good degree of quality; *Cl*OO showed the lowest peroxide value (7.9 mEq O_2_/kg oil); peroxides play a key role in cellular physiology due to their capacity to affect DNA, protein and lipid functioning [24,43]. The spectrophotometric values K_232_ and K_270_ (specific extinction in the UV at 232 and 270 nm), mainly related to the secondary products formed in the oxidation process, are within the legal limit for all the samples showed value, as well as the ∆K value, which expresses the difference between absorbance at 270 nm and 266 nm–274 nm [26,44]. 

#### 3.1.2. Phytochemical Composition 

Chlorophylls and carotenoids are strictly related to olive oil color, a quality parameter which can influence consumer choice and at the same time can be linked to olive variety, processing treatments as well as to storage conditions [24,45,46,47]. These two pigments are also very important for their probable beneficial effects on human health [47]. The results regarding total carotenoid content were significantly different among the three oils (Table 5). 

As expected, *Ca*OO exhibited the greatest carotenoid content, which was almost twofold higher than *Cl*OO. Considering the other family of oil pigments and the total chlorophyll, the three oils showed significant differences; notably the highest concentration of chlorophyll was observed in the CEVOO, while the chlorophyll concentration of the *Citrus* olive oils ranged from 7.06 mg/kg (*Cl*OO) to 6.47 mg/kg (*Ca*OO).

The health-promoting features of olive oil are the results of its specific composition including a balanced content of monounsaturated and polyunsaturated fatty acids and minor components, like phenolic compounds and tocopherols, which are both important for the nutritional quality of the product, as well as for its sensory profile and shelf life [46]. Thereafter, tocopherol profile and phenolic content of the *Citrus* olive oils were both determined.

In the experimental conditions considered, the addition of *Citrus* cryomacerated peels did not show any significant modification among oil samples, neither in terms of quantity of total tocopherols, nor considering the different isoforms, alpha, gamma, and delta.

Concerning the total phenol content, no significant differences were recorded among the sample, with value ranging around 140 ppm of gallic acid. Nevertheless, both *Ca*OO and *Cl*OO showed lower concentrations of hydroxityrosol and tyrosol than the CEVOO. These data are confirmed by the free-radical scavenging capacity (FRSC). Indeed, the *Citrus* olive oils showed a lower value than the control one in both analytical methods. 

The phenolic composition of olive oil affects its sensory properties too, because some phenols, (i.e., the secoridoid derivatives, such as oleuropein, and the ligstroside derivates) are thought to determine the bitterness and pungency of olive oil [48], therefore the compounds associated with olive oil bitterness have also been evaluated (Intensity of Bitterness, IB). According to Table 5, *Ca*OO showed the highest IB (0.84), together with the Control, while the lemon peel olive oil was significantly lower (IB = 0.48). 

**Figure 2:**
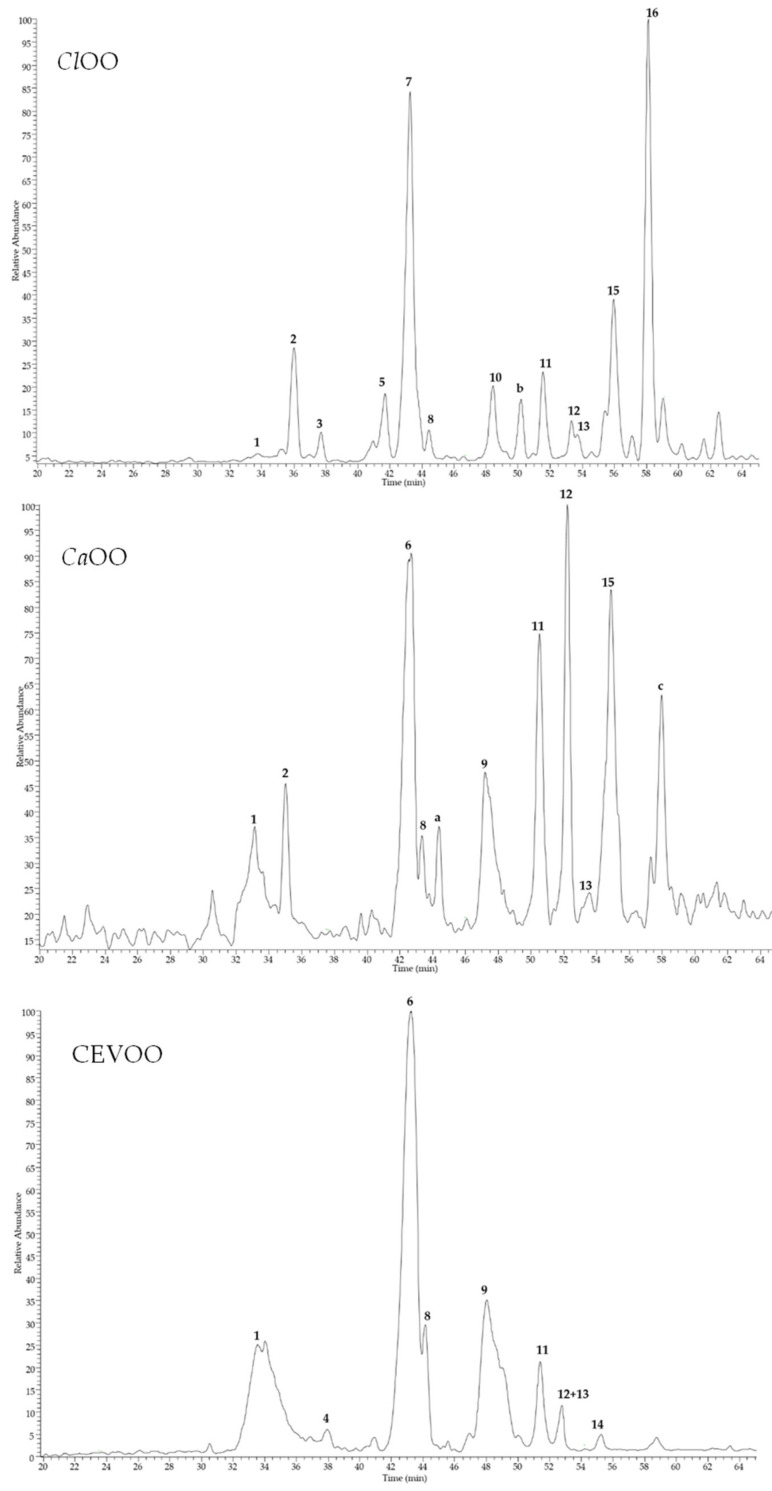
HPLC-ESI-MS/MS profiles, registered in negative ion mode, of control (CEVOO), *Citrus x aurantium* peel (*Ca*OO), and *Citrus limon* peel (*Cl*OO) olive oil extracts. Data of peaks 1–16 are listed in Table 6. Peaks a–c remained unidentified.

Control and *Citrus* EVOO extracts were also analysed by HPLC coupled with a photodiode array (PDA)/UV and electrospray ionization (ESI) mass spectrometer (MS). Chromatograms generated by MS detector in negative ion mode are shown in Figure 2.

The LC-MS profile of CEVOO showed the presence of nine compounds identified as elenolic acid (**1**), oleacein (**4**), hydroxytyrosol acyclodihydroelenolate (**6**), oleacin isomer (**8**), oleuropein aglycone (**9**), luteolin (**11**), oleuropein aglycone isomer (**12**), ligstroside aglycone (**13**), and apigenin (**14**). These results are in agreement with previous studies of phenol content of EVOO [49,50,51]. Compared to CEVOO, LC-MS analyses of *Citrus* EVOO extracts revealed some differences, especially due to the enrichments of molecules deriving from sour orange and lemon peels. In particular, in the *Ca*OO extract a significant intense peak (compound **15**) was observed, that was identified as diosmetin, a methoxyflavone previously reported in citrus peel [52]. Compound **15** was detected also in the *Cl*OO extract, together with other representative peaks. Indeed, *Cl*OO extract exhibited a more complex profile compared to *Ca*OO, with five major peaks attributable to limonoid aglycones and identified as limonexic/isolimonexic acid (**3**), citrusin isomers (**5** and **7**), limonin (**10**), and nomilinic acid (**16**). Limonoids are tetranortriterpenoids found in citrus seeds, pulp, and peels, and known as bitter compounds [53]. According to their polarity, limonoid aglycones occur mostly in *Citrus* peel, while glycoside forms are most abundant in pulp [19]. All compounds were tentatively identified by comparison of their elution order, UV, full MS, and fragmentation pattern (MS/MS) data (Table 6) with those reported in the literature.

Among constituents detected in all samples, compounds 1, 2, 4, 6, 8, 9, 12, and 13 were secoiridoids, while 11, 14 and 15 were identified as flavone aglycones, and 3, 5, 7, 10, and 16 were limonoids in aglycone form. Compound 1 (*t*_R_ = 33.5 min), detected in all samples as a broad peak but only in traces in *Cl*OO, showed λ_max_ at 248 nm and a deprotonated molecular ion [M-H]^−^ at *m*/*z* 241. MS/MS experiments generated a base ion peak at *m*/*z* 139 and other diagnostic product ions at *m*/*z* 209 and 165, according to data previously reported for elenolic acid [50]. Compound 2 (*t*_R_ = 36.0 min), detected in *Ca*OO and in *Cl*OO, differed from 1 for the presence of a hydroxyl group ([M − H]^−^ at *m*/*z* 257). Fragmentation pattern of the deprotonated molecule showed typical product ions at *m/z* 181 and 137, leading to identify 2 as hydrohylated elenolic acid [50]. Another elenolic acid derivative was detected in CEVOO and *Ca*OO extracts. Peak 6 (*t*_R_ = 43.0 min) had molecular weight 382 u, as indicated by deprotonated molecule [M − H]^−^ at *m*/*z* 381. Product ion at *m*/*z* 349 (base peak ion), generated by the loss of a CH_3_OH molecule, and other product ions at *m*/*z* 363, 245, and 151, is consistent with the fragmentation pattern of hydroxytyrosol acyclodihydroelenolate [54]. Compound 8 (*t*_R_ = 44.1 min), present in all analyzed oil samples, was characterized by a deprotonated molecule [M-H]^−^ at *m*/*z* 319, whose fragmentation pattern showed diagnostic peak at *m*/*z* 199 due to the loss of hydroxythyrosol unit, observed for oleacine [50]. An isomer of oleacine, presenting same MS and MS/MS data, was detected in a very small amount only in CEVOO (compound 4, *t*_R_ = 37.9 min, [M − H]^−^ at *m*/*z* 319). These isomers are probably due to open and closed ring forms [49]. Similarly, compounds 9 (*t*_R_ = 48.0 min) and 12 (*t*_R_ = 52.8 min) showed the same deprotonated molecule [M − H]^−^ at *m*/*z* 377; MS/MS of 9 evidenced diagnostic peaks at *m*/*z* 345, 307, and 275 typical of oleuropein aglycone in carboxylic form [50], while fragmentation pathway of 12 was characterized by a base ion peak at *m*/*z* 333 and product ions at *m*/*z* 301 and 181, previously reported for an oleuropein derivative as probable fragments derived from rearrangement [49]. Full MS of peak 13 indicated 362 u molecular weight ([M − H]^−^at *m*/*z* 361), suggesting the presence of a ligstroside aglycone, as confirmed by characteristic product ions at *m*/*z* 317 (due to the loss of carboxylic unit), 291 (base ion peak), and 259, in agreement with previous MS data [49]. In addition to secoiridoids, three flavone aglycones were detected in the analyzed oil samples, as deduced by UV absorptions at 267–268 and 342–352 nm. Full MS scan of compounds 11 (*t*_R_ = 51.1 min) and 14 (*t*_R_ = 55.0 min) showed deprotonated molecules [M − H]^−^ at *m*/*z* 285 and 269, respectively. MS/MS of both parent ions are in agreement with fragmentation profile of luteolin and apigenin, respectively [55]. Both flavones were previously reported in olive oil [49,56]. Peak 15 (*t*_R_ = 54.9 min) was detected only in *Citrus* oil samples, suggesting it originated from *Citrus* peels. Compound 15 had molecular weight 300 u, as indicated by full scan MS ([M − H]^−^ at *m*/*z* 299). MS/MS experiments produced a base peak ion at 284 u, due to the loss of a methyl group, suggesting 15 to be diosmetin, a methoxy flavone luteolin derivative, isolated from *Citrus* peel [52]. The chromatogram of *Cl*OO exhibited five peaks (3, 5, 7, 10, and 16) that were not found in the other samples typical of lemon peel. All compounds had close molecular weight and similar fragmentation patterns in the MS/MS experiments, suggesting they belonged to the same class. Compound 3 (*t*_R_ = 37.7 min) showed a deprotonated molecule [M − H]^−^ at *m*/*z* 501, together with product ions at *m*/*z* 457 and 413, due to the consecutive losses of two carboxylic units ([M − H-44-44]^−^). Thus, compound 3 was tentatively identified as limonexic acid or its constitutional isomer isolimonexic acid [57]. Compounds 5 (*t*_R_ = 41.7 min) and 7 (*t*_R_ = 43.2 min) had the same molecular weight 546 u and superimposable fragmentation pathways with ions at *m*/*z* 501, 457 and 397, generated by the successive losses of two CO_2_ molecules and an acetyl group, suggesting 5 and 7 to be two isomer forms of citrusin [19]. Compound 10 (*t*_R_ = 48.4 min) was revealed as formate adduct [M + HCOO]^−^ at *m*/*z* 51 whose fragmentation generated a base ion peak at *m*/*z* 469 due to the loss of a formic acid, thus was tentatively identified as limonin [14]. Full mass spectrum of peak 16 showed a deprotonated molecule [M − H]^−^ at *m*/*z* 531 and ion fragments at *m*/*z* 471 and 427 generated by the loss of an acetyl and then of a carboxyl group. These data led us to identify 16 as nomilinic acid [14]. Compounds 3, 5, 7, 10, and 16 are limonoids in aglycone form, common in citrus peel and seeds [19]. Few peaks remained unidentified. Peaks a (*t*_R_ = 44.4 min, [M − H]^−^ at *m*/*z* 201) and c (*t*_R_ = 58.0 min, [M-H]^−^ at *m*/*z* 329) were detected only in *Ca*OO, while peak b (*t*_R_ = 50.1 min, [M − H]^−^ at *m*/*z* 301) was found in *Cl*OO. Based on the analysis of mass fragmentation patterns, these molecules do not appear related to secondary metabolites commonly found in *Citrus* peel or in olive oil, thus they remained unidentified.

#### 3.1.3. Sensory Profiles

As reported in Table 7, the differences highlighted among the three olive oils (CEVOO, *Ca*OO, *Cl*OO) were significant for most of the considered parameter.

These data showed statistically significant differences among samples due to the addition of cryomacerated citrus peels to the olives. In particular the clarity of *Ca*OO and *Cl*OO was significantly higher than the control. The addition of peels also influenced the shades of the color; in particular, the *Ca*OO exhibited a higher value for the yellow shades, as expected considering its carotenoid content (Table 7). 

The smell profile was affected by the treatment too, especially in terms of detected descriptors: the *Ca*OO was characterized by a strong orange hint, while the *Cl*OO exhibits a strong scent of lemon. According to the legal limits for EVOO [26], no defects were detected by the panel and all the samples obtained a good score for the fruity features, thus confirming their good quality (Table 7). 

The taste parameters highlighted a higher level of bitter and pungent taste in both *Citrus* olive oils as well as a higher score for persistence. The bitter perception of the samples could be due not only to the secoiridoid derivatives content, evaluated by the IB (see Section 3.1.2), but also to the limonoid content, which reached the highest value in the *Cl*OO.

### 3.2. Nutraceutical Features of Citrus Olive Oils

#### 3.2.1. Effects of CEVOO, *Ca*OO and *Cl*OO Supplementation on Cardiometabolic Profile in HFD-Fed Rats

**Figure 3:**
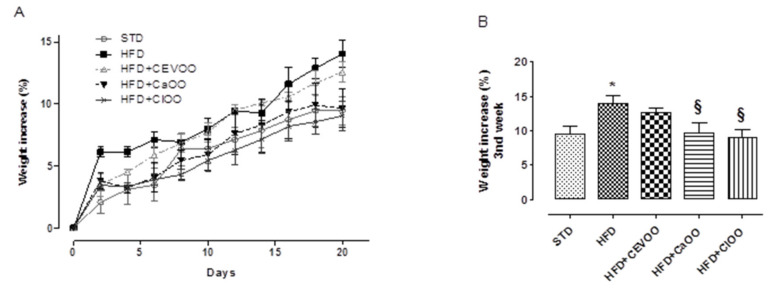
(**A**) Time course of the weight gain of animals during three weeks of treatment. (**B**) Final weight gain of the animals at the third week of treatment (end of treatment). * indicates a statistically significant difference between the HFD group and the STD group. § indicates statistically significant difference between HFD and HFD + CEVOO or HFD + *Ca*OO or HFD + *Cl*OO. Single symbol corresponds to *p* < 0.05.

A diet containing high levels of saturated fats contributes to significantly increasing body weight. Indeed, in these experimental conditions, animals fed with STD for three weeks showed a body weight percentage increase of 9.5 ± 1.2%, while animals fed with the HFD showed an increase of 14.0 ± 1.1%. Animals with CEVOO supplemented to the HFD showed a slightly reduced (not statistically significant) gain in body weight (12.6 ± 0.8%). In contrast, at the third week, animals fed with HFD + *Ca*OO and HFD + *Cl*OO showed a marked and significant containment of body weight gain (9.6 ± 1.5% and 9.0 ± 1.2%, respectively), superimposable with that observed in animals fed with STD (Figure 3).

According with the literature, HFD represents a well-known experimental model diet to induce an alteration of important metabolic parameters in rodents. In fact, beside the effects on body weight, this diet contributes to the generation of an increase in blood glucose, total cholesterol, LDL cholesterol and triglycerides, and a marked reduction in HDL levels [58,59]. 

Animals supplemented with CEVOO showed a marked reduction in blood glucose levels bringing them back to the standard values. *Cl*OO and *Ca*OO supplementation presented hypoglycemic effects; in particular C*l*OO, though not significantly, exhibited the highest value potency (Table 8).

Total cholesterol levels were markedly increased in animals fed with HFD. The addition of CEVOO modestly contributed to the lowering of total cholesterol levels, while both the *Citrus* enriched OOs were endowed with more evident hypo-cholesterolemic properties (Table 8).

A similar trend was observed for LDL levels, while no improvement was highlighted about HDL values. Therefore, the reduction in cardiovascular risk value (Total Cholesterol/HDL Cholesterol) reflected the effects of supplementation on total cholesterol (Table 8). Finally, the addition of CEVOO as well as *Ca*OO and *Cl*OO markedly reduced plasma triglycerides (Table 8).

HFD promoted a significant increase in liver weight; furthermore, the macroscopic analysis highlighted the presence of numerous fat deposits at this level. On the other hand, treatment with the oils did not show any significant effect on this parameter.

Other organs, such as the heart, did not show any significant weight change associated with HFD. 

**Figure 4:**
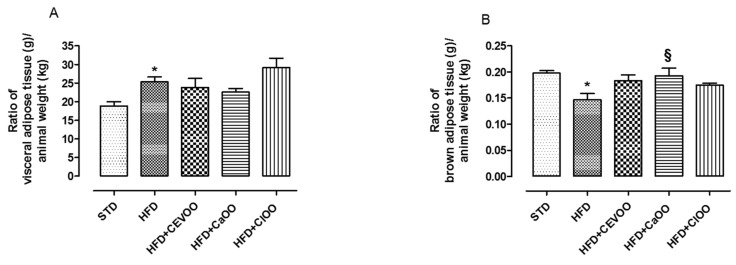
(**A**) Ratio of adipose tissue (g) in relation to animal weight (kg) after three weeks of treatment with different diets. (**B**) Ratio of brown adipose tissue (g) in relation to animal weight (kg) after three weeks of treatment with different diets. * indicates a statistically significant difference between the HFD group and the STD group. § indicates statistically significant difference between HFD and HFD + CEVOO or HFD + *Ca*OO or HFD + *Cl*OO. Single symbol corresponds to *p* < 0.05.

Visceral adipose tissue increased in the group fed with HFD, while the experimental treatments did not significantly influence this parameter (Figure 4A). Conversely, HFD reduced the amount of brown adipose tissue as already reported in the literature [60] (Figure 4B). The addition of CEVOO to the HFD increased the amount of brown adipose tissue, and *Cl*OO and *Ca*OO showed a similar effect (Figure 4B).

**Figure 5:**
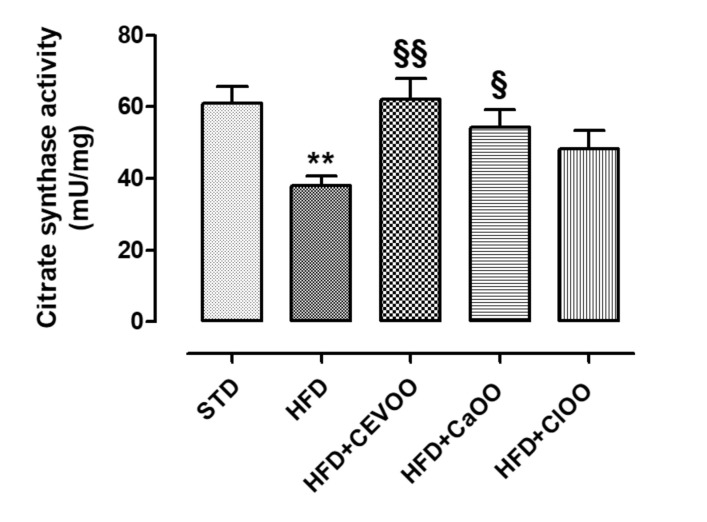
Activity of the enzyme citrate synthase in the adipose tissue. * indicates a statistically significant difference between the HFD group and the STD group. § indicates a statistically significant difference between HFD and HFD + CEVOO or HFD + *Ca*OO or HFD + *Cl*OO. Single symbol corresponds to *p* < 0.05; double symbol to *p* < 0.01.

Moreover, the activity of the citrate synthase enzyme was measured in adipose tissues of animals under different diets, as a marker of the metabolic activity of adipocytes (Figure 5). As previously described in the literature, the HFD significantly reduced the activity of citrate synthase [59], while the addition of EVOO brought it back to standard levels. The addition of *Ca*OO and *Cl*OO increased the average activity of this enzyme but less markedly than CEVOO (Figure 5).

**Figure 6:**
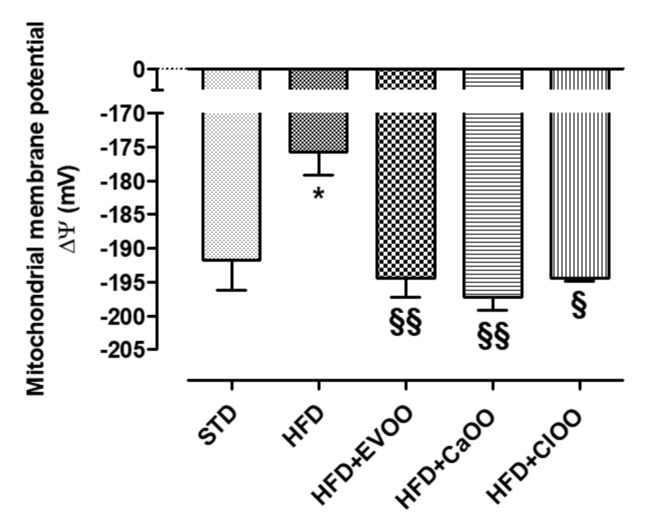
Changes in mitochondrial membrane potential in different animals. * indicates a statistically significant difference between the HFD group and the STD group. § indicates statistically significant difference between HFD and HFD + CEVOO or HFD + *Ca*OO or HFD + *Cl*OO. Single symbol corresponds to *p* < 0.05; double symbol to *p* < 0.01.

Finally, the myocardial tissue was used to evaluate the mitochondrial membrane potential. The mitochondrial membrane potential is a parameter closely correlated with the metabolic efficiency of heart mitochondria and therefore with their ability to produce ATP and tolerate “nocive insults”, as can be relied on in animals with a condition like metabolic syndrome. Normal ΔΨ is about −190 mV in a healthy animal, and the HFD appeared to be significantly depolarized at −175 mV (Figure 6). The addition of CEVOO reported the ΔΨ to the standard values and *Ca*OO and *Cl*OO in similar ways, suggesting that all three oils can effectively control the mitochondrial dysfunction associated with diet (Figure 6). 

#### 3.2.2. Effects of CEVOO, *Ca*OO and *Cl*OO Supplementation on Aortic Tissue Levels of Oxidative Stress Markers and Endothelial Function

##### Levels of Inflammatory and Oxidative Stress Markers 

**Figure 7:**
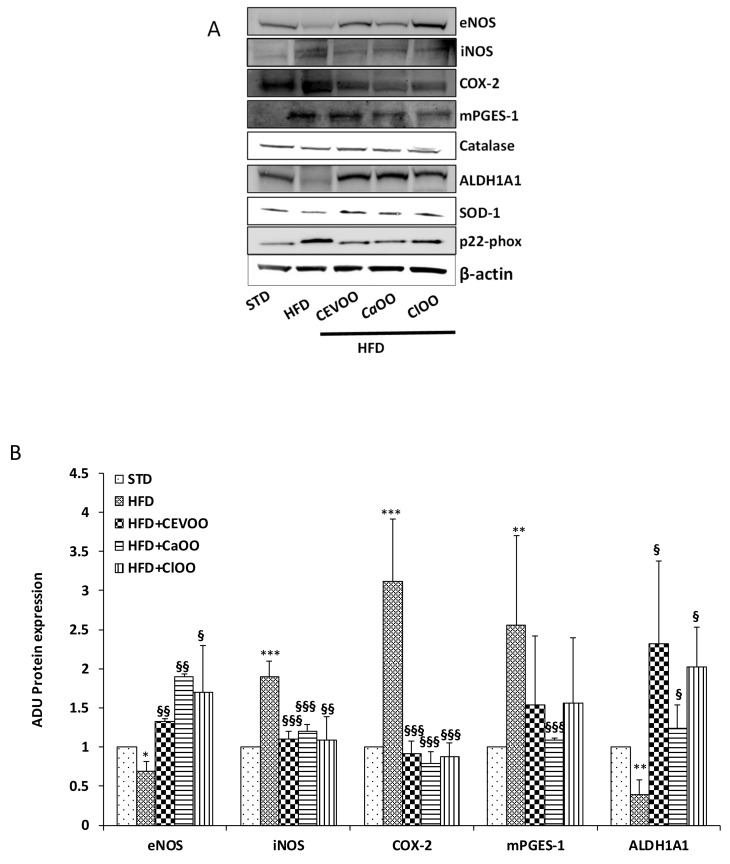
Effects of CEVOO, *Ca*OO and *Cl*OO diet supplementation on homeostatic, inflammatory and oxidant factors in aortic vessel tissues of HFD fed rats. (**A**) Western blot analysis of homeostatic factors (eNOS, ALDH1A1), inflammatory factors (iNOS, COX-2 mPGES-1) and oxidant factors (Catalase, SOD-1 and p22phox) in aortic vessel tissues. Each line contains 50 μg of total proteins obtained from aortic vessels of rats treated as designated. (**B**) Quantification of western blots. Data are reported as a ratio of arbitrary density unit (ADU) target protein/β-actin and are means ± standard deviation (*n* = 3); Data are reported as fold change vs. STD which was assigned to 1. * *p* < 0.05, ** *p* < 0.01, *** *p* < 0.001 vs. STD; § *p* < 0.05, §§ *p* < 0.01, §§§ *p* < 0.001 vs. HFD.

**Figure 8:**
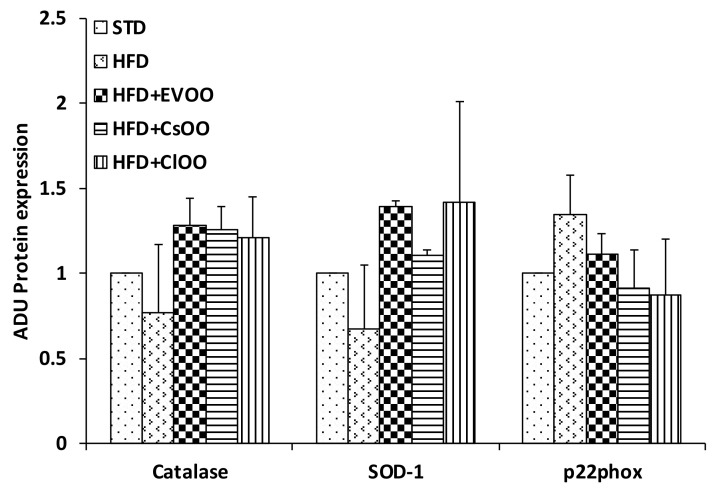
Effects of CEVOO, *Ca*OO and *Cl*OO diet supplementation on oxidant factors in aortic vessel tissues of HFD fed rats. Quantification of western blots for Catalase, SOD-1 and p33phox reported in Figure 7, panel A. Data are reported as a ratio of arbitrary density unit (ADU) target protein/β-actin and are means ± standard deviation (*n* = 3). Data are reported as fold change vs. STD which was assigned to 1.

**Figure 9:**
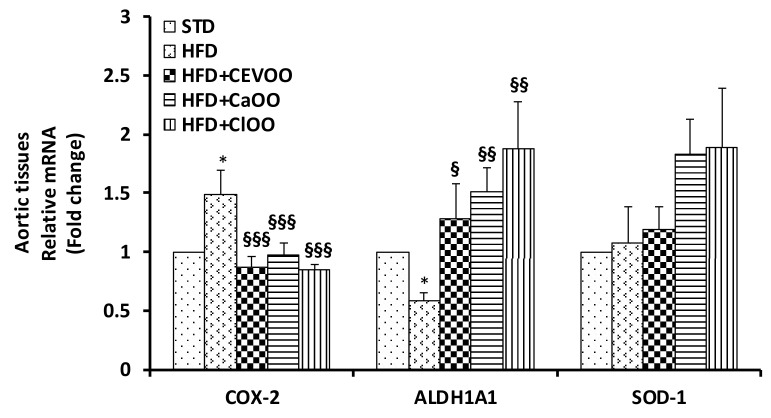
mRNA expression of COX-2, ALDH1A1 and SOD-1 in aortic vessel tissues of rats fed with HFD with/without CEVOO, *Ca*OO and *Cl*OO. Quantitative PCR analysis of COX-2, ALDH1A1 and SOD-1 in aortic vessel tissues. Data are reported as fold change expression, determined by the comparative *C*_t_ method (ΔΔ*C*_t_) normalized to GAPDH expression, and are means ± standard deviation (*n* = 3). Data as reported ad fold change vs. STD group which was assigned to 1; * *p* < 0.01 vs. STD; § *p* < 0.05, §§ *p* < 0.01, §§§ *p* < 0.001 vs. HFD.

Maintenance of blood vessel integrity and function is an active process, mediated by the balance among several homeostatic, inflammatory and oxidant factors. In this study, no significant change of blood pressure was observed in the treatment groups; indeed, STD-fed group showed a pressure value superimposable with HFD-fed group (data not shown). Probably, longer treatment times are required to highlight functional alterations at vascular level. However, the rat aortic tissues from all groups deriving from three weeks treatment were used to measure the protein expression and mRNA levels of endothelial nitric oxide synthase (eNOS), inducible NOS (iNOS), cyclooxygenase-2 (COX-2), microsomal prostaglandin E synthase-1 (mPGES-1), catalase, superoxide dismutase-1 (SOD-1), p22phox, and aldehyde dehydrogenase 1A1 (ALDH1A1) via Western blot and qPCR (Figure 7, Figure 8 and Figure 9). Compared with the STD group, the HFD significantly declined eNOS and ALDH1A1 protein levels, and increased the expression of iNOS, COX-2 and mPGES-1 in rat aortic tissues (Figure 7A,B). CEVOO supplementation in the HFD reverted eNOS, ALDH1A1, iNOS and COX-2 expression levels, thus reducing the inflammatory profile of the tissues known to contribute to the vascular dysfunction. Together the data suggests that constituents from orange and lemon supplemented into EVOO showed protective effects similar to those already shown for EVOO (Figure 7A,B) [61].

On the contrary, catalase, SOD-1 and p22-phox protein levels were not significantly affected by HFD nor by HFD supplemented with CEVOO, *Ca*OO and *Cl*OO (Figure 7A and Figure 8 for quantification). Of note, although not significant, we observed a trend of decreased expression for the two antioxidant enzymes, catalase and SOD-1, and increased expression for the most relevant source of reactive oxygen species, p22phox in HFD samples, while the addition to HFD of CEVOO or *Ca*OO or *Cl*OO reverted this trend. 

Consistently, at transcription level, we observed an increase of COX-2 and a decrease of ALDH1A1 in aortic tissues of rats fed with HFD, which were reverted by the supplement with EVOO, *Ca*OO or *Cl*OO. Expression of SOD-1 did not appear significantly affected in response to any dietetic treatments in rat aortic tissues (Figure 9). 

**Figure 10:**
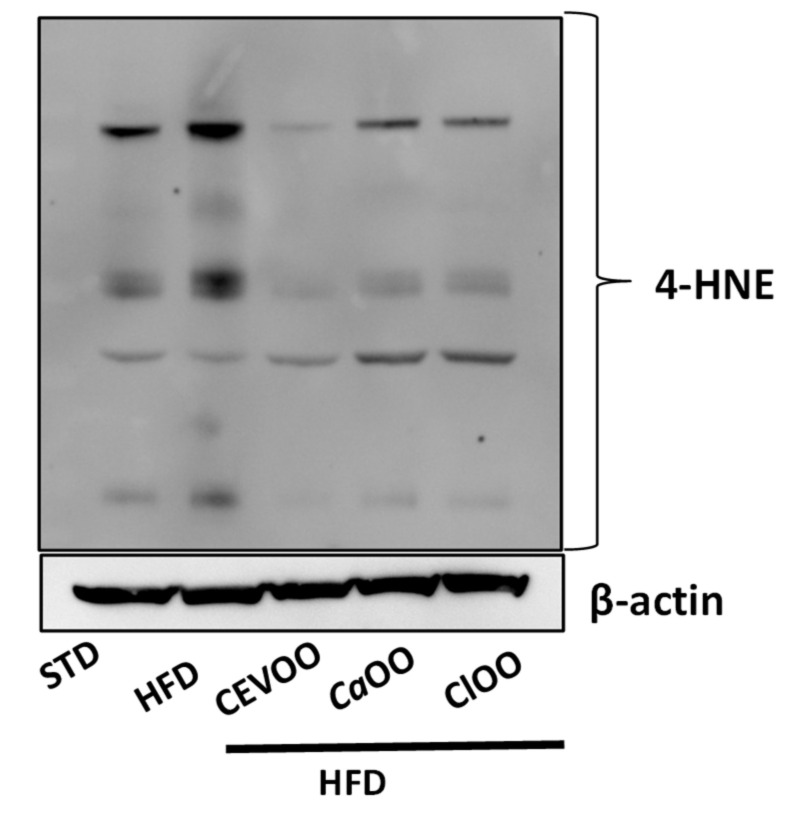
Effects of CEVOO, *Ca*OO and *Cl*OO diet supplementation on 4-HNE protein adducts in aortic tissues from HFD fed rats. The 4-HNE antibody recognizes the total of 4-HNE protein adducts. Representative western blot analysis of 4-HNE protein adducts. Each line contains 50 μg of total proteins obtained from aortic vessels of rats treated as designated. β-actin is reported as loading control.

However, although no significant changes in enzymes involved in the control of redox status in vascular tissues were observed, the HFD increased protein levels with adducts of 4-HNE, a product of lipid peroxidation, while CEVOO, *Ca*OO and *Cl*OO significantly reduced the 4-HNE-protein adducts, suggesting an antioxidant effect of the diet at the vascular level (Figure 10).

##### Endothelial Function

**Figure 11:**
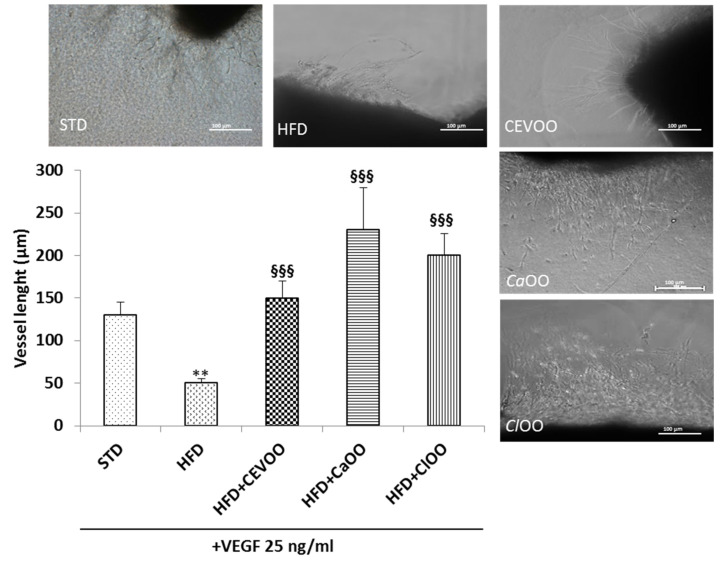
CEVOO, *Ca*OO and *Cl*OO promote vessel sprouting from aortic rings of HFD fed rats. Vessel sprouting from rat aorta rings at day 3 of incubation: all groups are treated with VEGF (25 ng/mL). Vessel length is expressed as the number of grid units covering the surface occupied by pseudo-capillaries in each well. *n* = 4 rats. ** *p*< 0.01 vs STD; §§§ *p*< 0.001 vs HFD. Each experimental point is run in triplicate.

HFD-induced oxidative stress has been associated with endothelial dysfunction [62]. In order to investigate the contribution of CEVOO, *Ca*OO and *Cl*OO on endothelial function in aortic rings from rats feed with HFD with/without EVOO, *Ca*OO and *Cl*OO, the endothelial cell sprouting activity was assessed. Aortic rings dissected from rats, deriving from treatment and cultured in a fibrin matrix, spontaneously generate outgrowths of branching micro-vessels [63]. HFD significantly impaired outgrowths of branching micro-vessels in response to the angiogenic factor VEGF 25 ng/mL (Figure 11). In aortic rings from EVOO, *Ca*OO and *Cl*OO, VEGF significantly promoted sprouting of micro-vessels, suggesting that the EVOO and olive oils enriched with citrus fruits, possessing a protective effect on endothelium, contributed to the maintaining of total blood vessel integrity and functionality (Figure 11).

##### Histological Assessment of CD40 in Aortic Vessel Tissue

**Figure 12:**
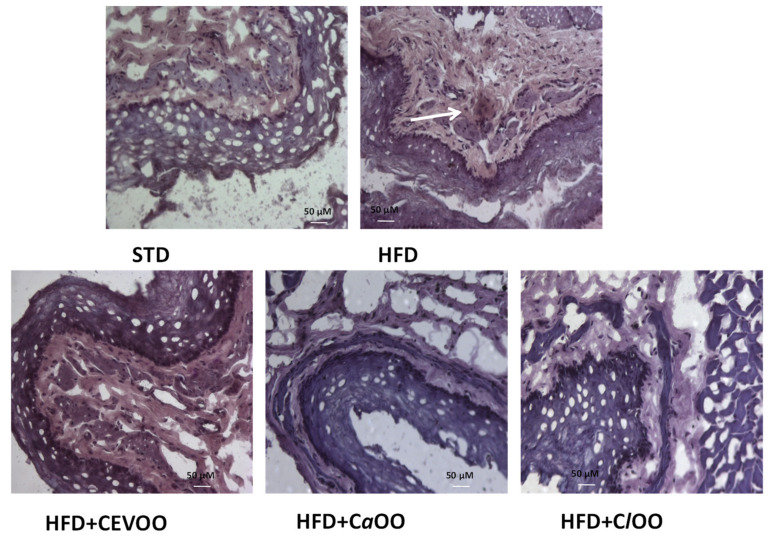
Effect of CEVOO, *Ca*OO and *Cl*OO supplementation on aortic vascular wall inflammation in HFD fed rats. Immunohistochemical evaluation of snap frozen sections from rat aortic tissues from STD, HFD, HFD + CEVOO, HFD + *Ca*OO and HFD + *Cl*OO. Arrow in HFD panel shows staining for CD40. One representative section is shown of three rats/experimental group. Scale bar 50 μM, original magnification ×60.

CD40 staining was performed to investigate the effects of CEVOO, *Ca*OO and *Cl*OO on inflammatory/immune cell infiltration in the aortic vascular wall. Photomicrographs of aortic vascular walls from all experimental groups revealed that expression of CD40 was absent in STD, CEVOO, *Ca*OO and *Cl*OO groups, while HFD group showed CD40 positive staining (Figure 12). 

## 4. Discussion

In our study, two fortified oils, namely *Ca*OO and *Cl*OO, obtained by cryomacerated peels of *Citrus* (*C. aurantium* and *C. limon*, *Ca*OO and *Cl*OO, respectively) added to olives during the oil extraction process were examined to determine whether the beneficial effects of EVOO, in terms of compositional and organoleptic properties as well as cardiovascular risk factors reduction, were maintained or eventually increased.

According to previous studies, if a cryogen, like dry ice (CO_2,s_), is added to a vegetal matrix, the intracellular water freezes [25,64,65]. Consequently, cellular membranes collapse, due to the density difference between ice and liquid water, thus cellular compounds spread in the liquid phase. Moreover, after sublimation the gaseous CO_2_ forms an inert gas layer under the vegetal material, protecting by oxidation the bioactive compounds as well as the essential oils present in citrus peels.

As we have previously reported [23,24], the use of cryomacerated *Citrus* by-products for the enrichment of olive oils seems to be useful also to improve the sensory profile of the oils, due to the ability of the cryomaceration technology, coupled to the cold pressing of olives, to preserve the peculiar aromatic scents of *Citrus* in the product, which exhibited a complex sensory profile, as expected considering the essential oil composition of *Citrus* peels. COOs could find application also as ingredient for innovative baking and pastry formulation, as they couple the peculiar scent coming from citrus peels to the technological and nutraceutical potential of extra-virgin olive oil.

The technological process used makes possible an enrichment of oils with biologically active substances, mainly from *Citrus* skin, such as carotenoids and phenols. Compared to Control (CEVOO), *Ca*OO exhibited a higher content of carotenoids (+81%), whose antioxidant properties have long been well known. As widely reported in the literature [18,66,67], a diet rich in carotenoid induces health benefits to cardiovascular apparatus and cognitive function and prevents some types of cancer and eye disease. Nevertheless, carotenoid intake via foods or supplements is not enough, especially in the elder population.

According to previous studies, LC-MS analyses of CEVOO evidenced that secoiridoids, such as oleuropein and ligstroside derivatives, were the most representative constituents that occur in the oil in the form of aglycons due to the hydrolysis of their glycosides during the oil extraction process [49]. Among phenol compounds, two flavone aglycons apigenin and luteolin were detected. Compared to CEVOO, *Ca*OO showed the presence of diosmetin, a flavone aglycone that usually can be found in orange peel also in the form of glycoside, such as diosmetin 6,8-di-*C*-glucoside [68]. The chemical profile of *Cl*OO was characterized by the presence of five typical *Citrus* limonoids in the form of aglycones, having a low polarity that can explain their presence in a non-polar medium such as olive oil.

Cardiovascular complications related to metabolic syndrome are increasing in various societies worldwide, and the consumption of a HFD is considered a major cause in the development of these complications [69,70]. In the present study, rats treated with a HFD developed dyslipidemia and became overweight, showed a significant reduction of adipocyte metabolism and myocardial mitochondrial functionality, and a significant increase of vascular inflammatory and oxidative stress factors, indicating this as a reliable model to study possible nutraceutical interventions on cardiovascular risk factors. 

According to the literature, a supplementation with EVOO contributes to ameliorating these parameters [71]; indeed, in our experimental conditions, CEVOO reduced plasma lipid levels in HFD-fed rats. The *Citrus* olive oils showed a similar profile on cardio-metabolic parameters and a more evident containment of the body weight gain (Table 8 and Figure 3). As regards the adipose tissue, a growing body of evidence points to the key role of brown adipose tissue in HFD-induced obesity [72]. Indeed, a reduction of brown adipose tissue has been reported in obesity conditions [60,72] and suggests that metabolic functions of these adipocytes can be partially compromised. Conversely, a transplantation of brown adipose tissue can promote the expression of various genes involved in fatty acid oxidation and raise protein expression of browning markers [72]. Of note, the supplementation with CEVOO increased the amount of brown adipose tissue, and a similar behavior was observed with *Cl*OO and *Ca*OO (Figure 4).

Further, as described in the literature, the HFD significantly reduced the activity of citrate synthase enzyme [59], while the addition of CEVOO brought it back to standard levels. *Ca*OO and *Cl*OO, although less markedly, increased the average activity of this enzyme (Figure 5). The improvement of citrate synthase activity underlies an improvement of metabolic activity of adipocytes, suggesting that nutraceutical supplementations developed in this work are able to positively influence this function. 

Finally, the mitochondrial dysfunction of myocardiocytes is closely related to metabolic syndrome. In this condition a lower metabolic efficiency of mitochondria, and therefore a reduced ability to produce ATP, can deeply compromise the tolerance of myocardium towards different kinds of pathological “insults” [73]. ΔΨ value is a useful parameter for evaluating the mitochondrial function. ΔΨ value was approximately −190 mV in STD-fed animals, while in HFD-fed animals the mitochondrial membrane was significantly depolarized (ΔΨ about −175 mV). 

Very interestingly, *Ca*OO and *Cl*OO, similarly to CEVOO, improved ΔΨ value in mitochondria obtained from cardiac tissues, suggesting that these oils are able to reduce myocardial dysfunction associated with this type of diet (Figure 6). 

No significant change of blood pressure was observed between STD and HFD diet. Probably, as reported in the literature, longer times of treatment should be attempted [74]. However, a nutraceutical approach is projected for preventing the progression of pre-pathological conditions and the cure of a pathological condition was not our objective; furthermore, despite the normotensive condition, pro-inflammatory markers and endothelial dysfunction in aortic vessels have been observed, thus we evaluated the effects of new fortified oils on these changes, prodromal of cardiovascular diseases.

With respect to blood vessels, the integrity and function of blood vessels plays a key role in the overall assessment of cardiovascular function. Indeed, the vascular wall represents the first front between the absorbed nutrients and the tissues of the body, and failure of this system results in many cardiovascular diseases such as atherosclerosis and tissue ischemia [61]. Excessive amounts of reactive oxygen species, known to exert a number of adverse effects on vessel function, may irreversibly inactivate protein kinases and/or phosphatases in the vascular wall affecting the barrier function of vessels [70,71,72]. Similarly, inflammation and the increase of inflammatory mediators in the blood vessel wall lead to the development and progression of cardiovascular diseases [73]. In this study, significant differences were observed with respect to inflammatory markers in the aorta tissues among groups treated with HFD and HFD + CEVOO, *Ca*OO or *Cl*OO. Indeed, in HFD fed rats, expression of COX-2, mPGES1, and iNOS was higher than in the standard diet fed rats, and the HFD + CEVOO, *Ca*OO or *Cl*OO groups showed a remarkable decrease of the inflammatory status of rat aorta, while increasing protective vascular markers, in line with other findings [75,76] (Figure 7A,B and Figure 9). Among the inflammatory mediators, the eicosanoids derived from the COX-2 and/or mPGES-1-mediated n-6 PUFA arachidonic acid metabolism, such as prostaglandin E2 (PGE2), and thromboxane, and expression of inducible nitric oxide synthase plays a key role in endothelial activation and trans-endothelial migration of monocytes into the vessel wall, two traditional risk factors for atherosclerosis. The inhibitory activity of EVOO, as well as of *Ca*OO or *Cl*OO, support a beneficial effect of olive oil-rich diets on inflammation and endothelial activation, and in particular, demonstrate that the addition of cryomacerated citrus peels to the olives do not alter the beneficial effects of olive oil.

No significant alterations were observed for enzymes involved in the control of redox state (Figure 7A, Figure 8 and Figure 9). Other studies have also come to similar conclusions with respect to EVOO performance on redox state in in vivo studies [77]. On the contrary, in both in vitro and in vivo studies, the phenolic extracts of EVOO have exhibited antioxidant effects [78]. Here, no clear effects were observed from CEVOO, *Ca*OO or *Cl*OO supplementation in HFD on antioxidant enzymes expression in aortic tissues, at least for the enzymes studied. However, expression of 4-HNE, a well-known by-product of lipid peroxidation endowed of atherogenic properties, widely accepted as a stable marker for oxidative stress, was higher in HFD group compared with HFD supplemented with oils, suggesting a functional and protective effect of the oils on the whole antioxidant profile of aortic tissues (Figure 10). In agreement with this observation, expression of ALDH1A1, an aldehyde scavenging enzyme, including 4-HNE, was significantly increased in aortic tissues of HFD + EVOO, *Ca*OO or *Cl*OO treated groups compared with the HFD group (Figure 7A,B and Figure 9).

Endothelial dysfunction, that is an important factor in the development and progression of cardiovascular diseases, leading to atherogenesis and plaque formation [79], has been studied with the rat aortic ring culture model [63]. Using this assay, we found that the HFD inhibited VEGF-induced endothelial cell sprouting, while supplementation of the diet with CEVOO, *Ca*OO or *Cl*OO reverted this inhibitory effect (Figure 11). The data suggest a protective activity of the three oils that might be associated with both their anti-inflammatory activity and their ability to modulate the lipid profile in serum of HFD fed rats, and therefore the endothelial integrity of the vessels [69]. Indeed, LDL, especially its oxidized form, promotes cytokine production from monocytes and endothelial cells, and on the surface of endothelial cells increases several adhesion molecules, including CD40 expression, associated with inflammation and endothelial dysfunction [80]. Further, CD40 decreases NO and increases ROS production by endothelial cells, inhibiting angiogenesis and contributing to the pathogenesis of cardiovascular diseases [80]. Immunohistochemical investigation confirmed the protective effects of CEVOO, *Ca*OO and *Cl*OO. In fact, while HFD increases CD40 expression in vascular aortic wall, the groups receiving the supplementation with the oils did not show any expression of CD40 (Figure 12).

Taken together, these results suggest that the enrichment of *Citrus* olive oils by means of their typical active metabolites, usually absent in the olive oils, possesses a superimposable profile compared to CEVOO, and sometimes an enhanced nutraceutical value, especially on body weight gain and lipidic profile.

## 5. Conclusions

*Citrus* peels can be considered as a source of bioactive compounds useful to improve the sensory profile as well as the phytochemical composition of olive oils. Indeed, we demonstrated that the technology adopted to produce *Citrus* oils, enriches them with functional and bioactive compounds typical of *Citrus* peel and furthermore improves their organoleptic properties without altering their beneficial effects. Indeed, like Control extra virgin olive oil, C*a*OO and C*l*OO showed protective effects on glucose and serum lipid levels, metabolic activity of adipocytes, myocardial tissue functionality, oxidative stress markers and endothelial function at blood vessel level.

## Figures and Tables

**Table 1:** Olive fruit characterization. Data are expressed as mean ± confidence interval (*n* = 3) at *p* = 0.05.

Parameter	
Maturity index (0:7)	4.1 ± 0.1
Dry Matter (%)	51.57 ± 0.03
Oil Content (% d.m.)	19.70 ± 0.04

**Table 2:** Sample codes used to identify the olive oil formulations developed.

Sample Codes	Description
CEVOO	Control Extra Virgin Olive Oil
*Ca*OO	Olive Oil obtained with 25% w/w of *Citrus aurantium* peels
*Cl*OO	Olive Oil obtained with 25% w/w of *Citrus limon* peels

**Table 3:** Composition of the two diets used: standard diet (STD) and high fat diet (HFD).

	STD	HFD
Protein (%)	14.3	12.9
Fat (%)	4.0	19.2
Carbohydrate (%)	48.0	50.2
Calories from Protein (%)	20.0	12.1
Calories from Fat (%)	13.0	40.7
Calories from Carbohydrate (%)	67.0	47.2
Na (mg/kg)	1000.0	2234.1
K (mg/kg)	6000.0	5284.3
Mg (mg/kg)	2000.0	1294.0
Ca (mg/kg)	7000.0	6312.5
Mn (mg/kg)	100.0	47.7
Fe (mg/kg)	175.0	253.2
Cu (mg/kg)	15.0	18.5
Zn (mg/kg)	70.0	48.7
P (mg/kg)	6000.0	5144.2
Cl (mg/kg)	3000.0	3508.1
Vitamin A (IU/g)	6.0	7.4
Vitamin E (IU/kg)	120.0	29.8
Vitamin D_3_ (IU/g)	0.6	1.0
Vitamin K_3_ (mg/kg)	20.0	13.3
Vitamin B_1_ (mg/kg)	12.0	4.2
Cholesterol (mg/kg)	-	12488

**Table 4:** Chemical characterization of the control (CEVOO), *Citrus x aurantium* peel olive oil (*Ca*OO), *Citrus limon* peel olive oil (*Cl*OO) and EVOO legal limits Regulation [26].

	EVOO [26]	CEVOO	*Ca*OO	*Cl*OO	Significance Level ^1^
Free acidity (% oleic acid w/w)	≤0.80	0.74 ^a^	0.60 ^b^	0.74 ^a^	**
Peroxide index (mEq. O_2_/kg oil)	≤20.00	8.90 ^a^	9.60 ^a^	7.90 ^b^	**
K_232_	≤2.50	1.98 ^c^	2.32 ^b^	2.39 ^a^	***
K_270_	≤0.22	0.14 ^b^	0.19 ^a^	0.18 ^b^	**
ΔK	≤0.01	0.00	0.00	0.00	n.s.

^1^ Significance level—*** *p* < 0.001; ** *p* < 0.01; n.s: not significant (*p* ≥ 0.05). Within the same row, parameters sharing the same letter do not have a significantly different mean value.

**Table 5:** Phytochemical characterization of control (CEVOO), *Citrus x aurantium* olive oil (*Ca*OO) and *Citrus limon* olive oil (*Cl*OO).

	CEVOO	*Ca*OO	*Cl*OO	Significance Level ^1^
Total carotenoid (ppm lutein)	4.62 ^b^	8.38 ^a^	4.09 ^c^	***
Total chlorophylls (ppm pheophytin)	9.93 ^a^	6.47 ^c^	7.06 ^b^	***
α-tocopherol (ppmVitamin E)	117	123	110	n.s.
γ-tocopherol (ppmVitamin E)	3.4	3.6	2.0	n.s.
δ-tocopherol (ppmVitamin E)	0.53	1.8	1.2	n.s.
Total phenols (ppm gallic acid)	144	140	139	n.s.
FRSC _ABTS_ (μmol TEAC/mL)	0.40 ^a^	0.30 ^b^	0.30 ^b^	***
FRSC _DPPH_ (μmol TEAC/mL)	0.34 ^a^	0.24 ^b^	0.26 ^b^	***
Hydroxtyrosol (ppm)	0.06 ^a^	0.02 ^c^	0.04 ^b^	***
Tyrosol (ppm)	2.7 ^a^	1.6 ^b^	1.2 ^c^	***
Intensity of Bitterness	0.85 ^a^	0.84 ^a^	0.48 ^b^	***

^1^ Significance level—*** *p* < 0.001; n.s: not significant (*p* ≥ 0.05). Within the same row, parameters sharing the same letter do not have a significantly different mean value.

**Table 6:** Chromatographic, UV, and ESI-MS/MS data of chemical constituents detected in control (CEVOO), *Citrus x aurantium* peel (*Ca*OO), and *Citrus limon* peel (*Cl*OO) olive oil extracts.

Peak *	Compound	*t*_R_ (min)	MW (u)	[M − H]^−^ (*m/z*)	[M + HCOO]^−^ (*m/z*)	MS/MS Ions **	UV	Sample
	*Secoiridoids*							
1	Elenolic acid (EA)	33.5	242	241		209, 165, 139, 127, 121, 101, 95	248	CEVOO *Ca*OO *Cl*OO
2	Hydroxylated elenolic acid	36.0	258	257		225, 181, 137	243	*Ca*OO *Cl*OO
4	Oleacein, isomer I (oleuropein aglycone decarboxymethyl or 3,4-DHPEA-EDA)	37.9	320	319	365	199	250	CEVOO
6	Hydroxytyrosol acyclodihydroelenolate (HT-ACDE)	43.0	382	381		363, 349, 335, 303, 285, 245, 151	239, 277	CEVOO *Ca*OO
8	Oleacin, isomer II	44.1	320	319	365	199, 181, 153, 111	250	CEVOO *Ca*OO *Cl*OO
9	Oleuropein aglycone (3,4-DHPEA-EA)	48.0	378	377		345, 307, 275, 149	247, 286	CEVOO *Ca*OO
12	Oleuropein derivative	52.8	378	377		333, 301, 181, 275, 149	249, 277	CEVOO *Ca*OO *Cl*OO
13	Ligstroside aglycone (*p*-HPEA-EA)	52.8	362	361		335, 317, 291, 259	276	CEVOO *Ca*OO *Cl*OO
	*Flavones*							
11	Luteolin	51.1	286	285		267, 241, 217, 199, 175, 151	267, 352	CEVOO *Ca*OO *Cl*OO
14	Apigenin	55.0	270	269		241, 225, 201, 183, 181, 151	268, 342	CEVOO
15	Diosmetin	54.9	300	299		284, 271, 227	268, 344	*Ca*OO *Cl*OO
	*Limonoids*							
3	Limonexic/isolimonexic acid	37.7	502	501		457, 413, 371, 279	215	*Cl*OO
5	Citrusin (isomer I)	41.7	546	545		501, 457, 397, 353	215	*Cl*OO
7	Citrusin (isomer II)	43.2	546	545		501, 457, 397, 353	215	*Cl*OO
10	Limonin ***	48.4	470	469	515	469, 427, 411	210	*Cl*OO
16	Nomilinic acid	58.1	532	531		489, 471, 427, 369, 307	210	*Cl*OO

* Peak number correspond to those showed in Figure 2. ** Product ions are produced by fragmentation of deprotonated molecules [M − H]^−^. *** Product ions were generated by fragmentation of [M + HCOO]^−^ adduct.

**Table 7:** Two-way ANOVA calculated for all the parameters evaluated by panelists during tasting sessions for the three olive oils, with the panelists and addition of citrus peels as main effects.

Quantitative Parameter		CEVOO	*Ca*OO	*Cl*OO	Significance Level ^1^
View	Clarity	3.80 ^b^	6.57 ^a^	6.43 ^a^	***
	Green shadows	2.41 ^ab^	1.20 ^b^	3.84 ^a^	**
	Yellow shadows	3.67 ^b^	6.10 ^a^	2.74 ^c^	***
Smell	Smell intensity	4.26	4.23	5.34	n.s
	Green fruity	0.27	0.33	0.44	n.s.
	Ripe fruity	3.01 ^a^	0.64 ^b^	1.14 ^ab^	**
	Cut grass	0.40	0.56	0.00	n.s
	Olive leaf	0.54	0.46	0.00	n.s
	Lemon	0.00 ^b^	0.00 ^b^	5.29 ^a^	***
	Orange	0.21 ^b^	3.17 ^a^	0.00 ^c^	***
	Candied fruit	1.36	1.39	2.64	n.s
	Other	1.71	0.00	0.86	n.s.
Taste	Bitter	0.33 ^c^	3.67 ^b^	4.66 ^a^	***
	Pungent	0.40 ^b^	3.29 ^a^	1.37 ^ab^	**
	Sweet	3.43 ^a^	0.27 ^b^	0.74 ^b^	***
	Aftertaste	0.49	0.00	0.93	n.s.
	Persistence	1.84 ^b^	4.09 ^a^	3.70 ^ab^	**
Defects	Heating	0.00	0.00	0.00	-
	Mold/humidity	0.00	0.00	0.00	-
	Winey/vinegary	0.00	0.00	0.00	-
	Sludge	0.00	0.00	0.00	-
	Metallic	0.00	0.00	0.00	-
	Rancid	0.00	0.00	0.00	-

**^1^** Significance level—*** *p* < 0.001 (F = 8.49); ** *p* < 0.01 (F = 5.09); n.s: not significant. Within the same row, parameters sharing the same letter do not have a significantly different mean value.

**Table 8:** Data of lipid and glycemic profiles observed after three weeks of treatment.

	STD	HFD	HFD + CEVOO	HFD + *Ca*OO	HFD + *Cl*OO
Total Cholesterol (mg/dL)	75.3 ± 3.1	111.4 ± 4.1 **	94.8 ± 7.1	73.5 ± 4.7 ^§§§^	81.7 ± 10.7 ^§^
Triglycerides (mg/dL)	77.3 ± 6.3	113.4 ± 13.4 *	64.4 ± 3.2 ^§^	72.8 ± 14.8 ^§^	68.7 ± 3.2 ^§^
Cholesterol HDL (mg/dL)	48.7 ± 2.9	22.3 ± 1.9 ***	23.2 ± 3.5	22.5 ± 3.1	21.3 ± 2.3
Cholesterol LDL (mg/dL)	31.7 ± 10.0	60.4 ± 5.2 *	57.3 ± 6.7	42.6 ± 6.3	46.7 ± 12.2
Cholesterol non-LDL (mg/dL)	26.5 ± 1.2	88.8 ± 3.6 ***	71.5 ± 6.3 ^§^	51.2 ± 5.2 ^§§§^	60.3 ± 12.1 ^§§^
Cardiovascular risk (Total Cholesterol/Cholesterol HDL)	1.6 ± 0.1	5.2 ± 0.4 ***	4.4 ± 0.6	3.4 ± 0.5	4.1 ± 1.0
Blood Glucose (mg/dL)	64.3 ± 4.8	79.4 ± 8.9	62.8 ± 4.1	67.8 ± 10.1	52.5 ± 11.5

* Indicates a statistically significant difference between the HFD group and the STD group. § indicates statistically significant difference between HFD and HFD + CEVOO or HFD + *Ca*OO or HFD + *Cl*OO. Single symbol corresponds to *p* < 0.05; double symbol to *p* < 0.01 and triple symbol to *p* < 0.001.
